# Role of PKD2 in Rheotaxis in *Dictyostelium*

**DOI:** 10.1371/journal.pone.0088682

**Published:** 2014-02-10

**Authors:** Wanessa C. Lima, Adrien Vinet, Jean Pieters, Pierre Cosson

**Affiliations:** 1 Department of Cell Physiology and Metabolism, Centre Médical Universitaire, University of Geneva, Geneva, Switzerland; 2 Biozentrum, University of Basel, Basel, Switzerland; University of Dundee, United Kingdom

## Abstract

The sensing of mechanical forces modulates several cellular responses as adhesion, migration and differentiation. Transient elevations of calcium concentration play a key role in the activation of cells following mechanical stress, but it is still unclear how eukaryotic cells convert a mechanical signal into an ion flux. In this study, we used the model organism *Dictyostelium discoideum* to assess systematically the role of individual calcium channels in mechanosensing. Our results indicate that PKD2 is the major player in the cell response to rheotaxis (i.e., shear-flow induced mechanical motility), while other putative calcium channels play at most minor roles. Mutant *pkd2* KO cells lose the ability to orient relative to a shear flow, whereas their ability to move towards a chemoattractant is unaffected. PKD2 is also important for calcium-induced lysosome exocytosis: WT cells show a transient, 2-fold increase in lysosome secretion upon sudden exposure to high levels of extracellular calcium, but *pkd2* KO cells do not. In *Dictyostelium*, PKD2 is specifically localized at the plasma membrane, where it may generate calcium influxes in response to mechanical stress or extracellular calcium changes.

## Introduction

Prokaryotic and eukaryotic cells are constantly exposed to mechanical forces, both extracellular (e.g. shear force exerted by a fluid flow, gravity, contact) and intracellular (e.g. changes in osmotic pressure), to which they respond by regulating many cellular processes, such as cell adhesion and migration, mitosis, gene expression, and cell differentiation [1], [2]. Mechanosensing involves the perception of mechanical forces and their conversion into intracellular biochemical signals [3], [4]. The intracellular response induced by mechanical stress depends largely on ion fluxes, particularly calcium fluxes caused by the opening of calcium channels [5], [6]. In bacteria, Msc (for mechanosensing) channels are directly gated by membrane deformations caused by changes in cell osmolarity, and initiate intracellular signaling [7]. However, to date no eukaryotic mechanosensing receptor and/or channel have been unambiguously identified [3], [8]. Rather, mechanosensing in eukaryotic cells is thought to involve the regulatory action of protein complexes linking the extracellular matrix (ECM) or the cytoskeleton to ion channels.

Several families of ion channels, mostly non-selective calcium channels from the TRP family [9], have been implicated in mechanosensing in *C. elegans*, *Drosophila* and mammals, but it is still not clear if they are directly or indirectly gated by mechanical stress [3], [10]. For example, early observations suggested that TRPC6 channel could be directly activated by changes in membrane tension, but recent findings rather indicate that this channel is indirectly activated by the angiotensin II type 1 receptor [11], [12]. TRPP2 (also named PKD2 or polycystin-2) is a calcium channel that forms a complex with PKD1, and the PKD1/PKD2 complex has been implicated in intracellular calcium increases in mechanically stressed ciliated cells [13]–[15]. However some studies indicate that the PKD complex may act rather by interacting with the cytoskeleton and regulating an as yet unidentified channel [16], [17]. In addition to TRP channels, metazoan candidates for mechanosensitive components include sodium channels of the ENaC family, two-pore domain potassium channels (K2P) and bacterial Msc-like channels [8], [18].

The amoeba *Dictyostelium discoideum* is a model organism easily amenable to genetic analysis, and largely used to study cell migration and chemotaxis, as the core mechanisms involved in motility are largely conserved from amoebae to human cells [19]. Several publications have reported that migration and physiology of *Dictyostelium* cells are modulated by mechanical stresses induced by a fluid flow, electrical fields or compression [20]–[23]. Remarkably, the total number of putative ionic channels is extremely reduced in *Dictyostelium* compared to other organisms. The *Dictyostelium* genome contains only three genes encoding putative calcium channels potentially expressed at the cell surface or in endocytic compartments (*mcln*, *pkd2*, *tpc*) as well as one Msc-like channel (*mscS*) [24], [25]. In addition, one IP_3_ receptor (*iplA*) is potentially present in the ER, and five P2X receptors (*p2xA-E*) are restricted to the contractile vacuole [26]. Since P2X receptors are thought to play a specific role in the function of the specialized osmo-regulatory contractive vacuole, they were not considered further in this study. The low number of channels and the relative ease with which specific knockout strains can be generated and analyzed makes *Dictyostelium* a unique system by allowing a systematic comparative analysis of the role of each channel in mechanosensing.

In this study, we generated specific knockout strains for the *mcln*, *pkd2*, *tpc*, *mscS* and *iplA* channels in *Dictyostelium* and characterized their role in rheotaxis (or shear-flow-induced cell motility). Our results reveal that PKD2 plays a key role in rheotaxis in *Dictyostelium* amoebae.

## Results

### Rheotaxis in *Dictyostelium*

The *Dictyostelium discoideum* genome exhibits a reduced number of genes encoding proteins potentially involved in mechanotransduction, including some ionic channels (MscS, IplA, PKD2, TRP-ML, and TPC2) and one integrin beta-like protein (SibA) (Table 1). To determine the role of these different proteins in mechanotransduction, we first tested the ability of WT and specific KO cell lines for each of these six genes to respond to shear-flow induced stress. For this, *Dictyostelium* cells were allowed to attach to a glass coverslip and their migratory behavior was assessed before and after the initiation of a uniform fluid flow (Figure S1 shows a schematic diagram of the flow chamber used).

**Table 1:** *Dictyostelium* orthologs with a potential role in mechanosensing.

Gene	Dictybase ID	UNIPROT accession	Global similarity to human ortholog	Reference
*mscS*	DDB_G0277253	Q54ZV3	43%*	This study
*sibA*	DDB_G0287363	Q54KF7	50%$	[46]
*iplA*	DDB_G0292564	Q9NA13	43%	This study
*mcln*	DDB_G0291275	Q54EY0	44%	[24]
*pkd2*	DDB_G0272999	Q558Y3	46%	This study
*tpc2*	DDB_G0289105	Q54HZ8	49%	This study

* Similarity to the *Arabidopsis thaliana* ortholog (no human ortholog exists for this protein).

$ Considering only the VWA motif (see original paper for more information).

**Figure 1:**
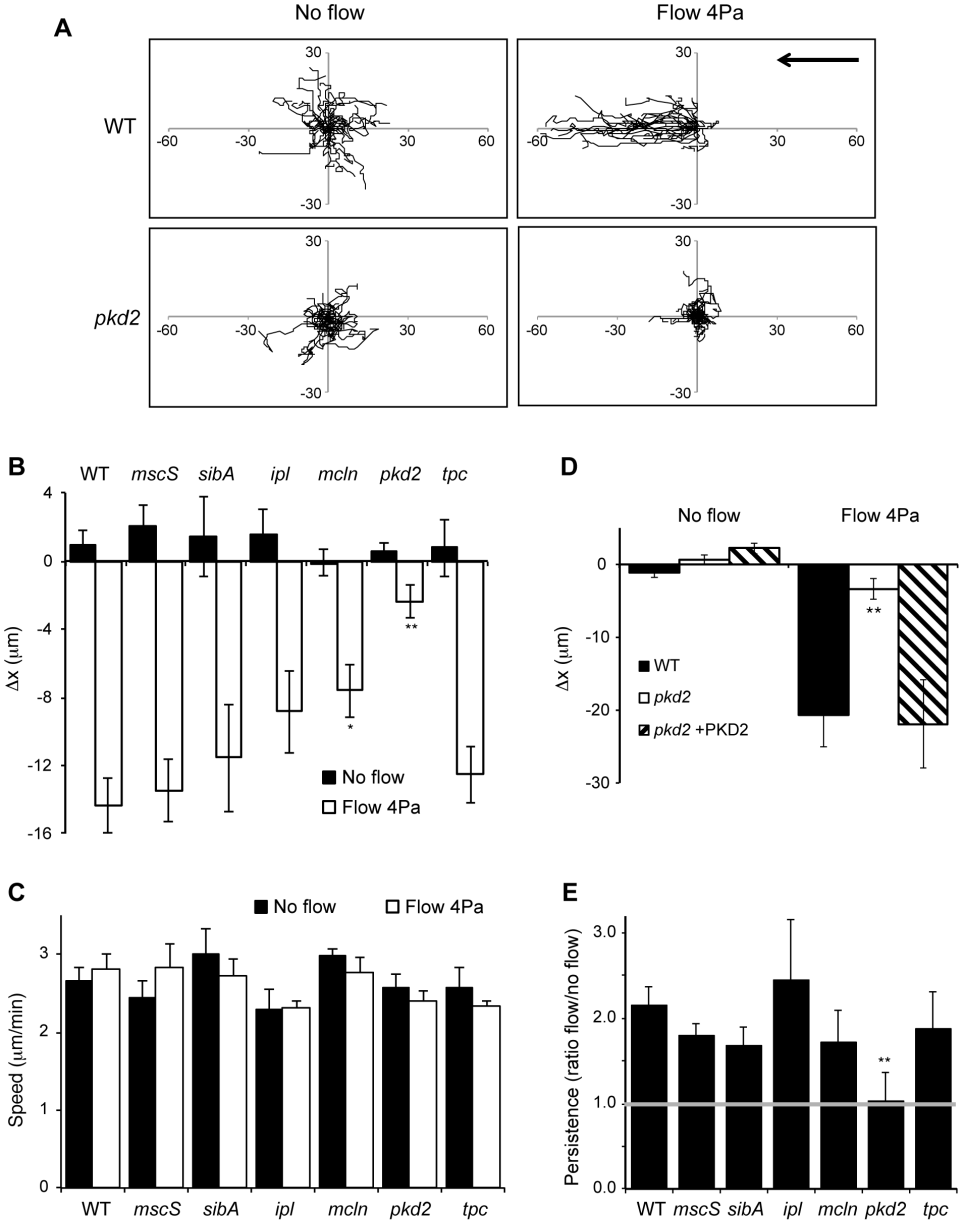
PKD2 is essential for shear-fluid stress response. A) Trajectories of WT and *pkd2* KO cells migrating randomly (no flow) or subjected to shear flow stress (flow 4 Pa); cells were imaged every 15 sec for a total of 10 min, and the origins were set to 0. Axes indicate distances in µm. The arrow indicates the direction of the flow (from right to left). One representative experiment is shown. B) The directionality of cell migration was assessed by measuring the net displacement on the X axis (Δx). No significant difference between WT and KO cells was seen in control (no flow) condition. Under shear stress, *mcln* KO cells showed a significant reduction in orientation, while *pkd2* KO cells were almost unable to orientate in the direction of the flow. * p<0.05, ** p<0.01, compared to WT values; n = 5. C) The migration speed was calculated as the total distance migrated divided by the time (µm/min). Speed was unchanged upon exposure to a shear stress, and no significant difference was seen between WT and KO cells; n = 5. D) Expression of PKD2 in *pkd2* KO cells restores the ability of cells to orientate relative to a shear flow. ** p<0.01, compared to WT values; n = 4. E) Persistence was measured as the net distance between initial and final cell positions divided by the total distance. Here it is shown the ratio between the persistence when cells migrate randomly and when exposed to a shear flow. Only *pkd2* KO cells did not show an increased persistence when submitted to a shear stress. ** p<0.01, compared to WT values; n = 5.

As reported previously [20], WT cells respond to shear stress by moving in the same direction as the fluid flow (Figure 1A, and Movies S1 and S2). To quantify this oriented movement, we measured the net displacement of cells on the X axis, parallel to the flow (Δx) (Figure 1B). In the absence of flow, both WT and KO cell lines migrated randomly (Δx close to 0) and with similar speed (around 2.5 µm/min) (Figure 1C). When exposed to a constant flow for 10 min (with an applied force equivalent to 4 Pa), WT cells moved at the same speed (Figure 1C) and oriented in the direction of the flow (from right to left, as indicated by negative Δx values) (Figure 1B). Of all the KO cell lines analyzed, only *pkd2* KO cells showed an almost complete loss of directionality when exposed to a flux (Figure 1B, and Movies S3 and S4). A WT phenotype was restored when *pkd2* KO cells were transfected with an expression vector harboring the PKD2 coding sequence (Figure 1D). *mcln* KO cells also showed a significant decrease in their response to shear stress, although not as pronounced as *pkd2* KO cells, and *iplA* KO cells showed a weak and not statistically significant decrease in directionality (Figure 1B). Orthologs of Msc and TPC2 channels and the beta-integrin-like SibA protein did not appear to be involved in response to mechanical stress in *Dictyostelium*.

Another way of analyzing the behavior of cells submitted to a shear stress is to determine their directional persistence. When submitted to a fluid flow, the directional persistence of WT cells increased two-fold, and the same happened for all the KO cells (Figure 1E). However, *pkd2* KO cells did not show any increase in persistence when submitted to fluid flow. These results indicate that the PKD2 channel plays a unique role in *Dictyostelium* mechanosensing, and this led us to further study its structure and localization.

### Structure and localization of PKD2

**Figure 2:**
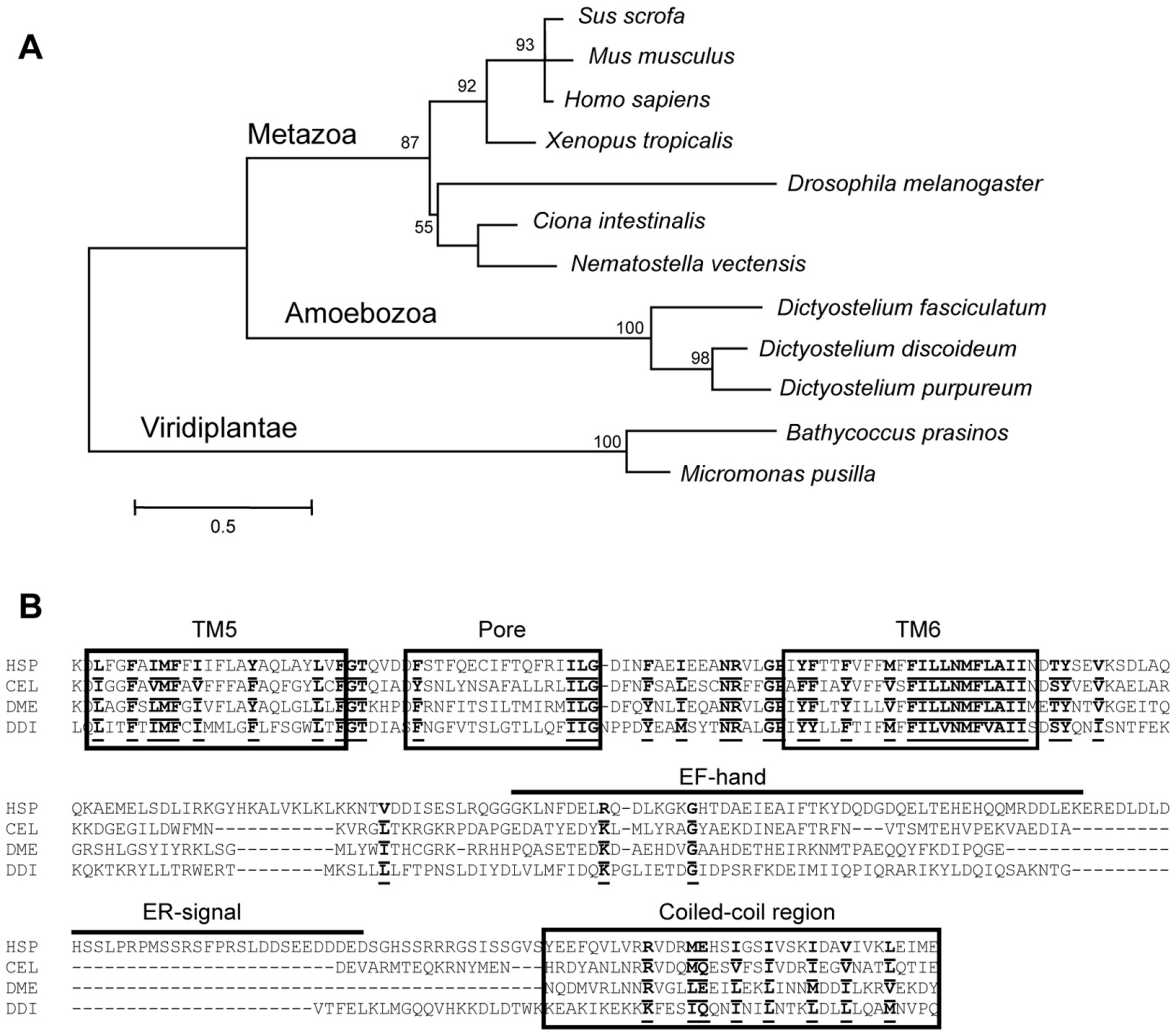
Phylogeny and primary structure of *Dictyostelium* PKD2. A) Unrooted maximum likelihood tree of the PKD2 family. Branch lengths are proportional to the number of amino acid substitutions. Numbers at the nodes represent the percentage of bootstrap support (only values >50% are shown). B) Protein alignment of the conserved C-terminal region, spanning TM domains 5 and 6, the pore and coiled-coil regions (boxed). Two other features, only present in the human ortholog, are also indicated: the EF-hand domain and a region essential for endoplasmic reticulum localization. Bold underlined residues are conserved in human (HSP, GenBank accession number NP_000288), *C. elegans* (CEL, NP_502838), *D. melanogaster* (DME, NP_609561) and *Dictyostelium discoideum* (DDI, XP_644933) orthologs. Gaps are denoted by broken lines.

*Dictyostelium* PKD2 belongs to the TRP (Transient Receptor Potential) family of ion channels, and phylogenetic analysis places it at the base of the Metazoan group (Figure 2A). It presents the distinctive features of the TRP family: six transmembrane (TM) domains, a conserved pore region between TM5 and TM6 and a large extracellular loop between TM1 and TM2 [27]. In addition, the C-terminal domain contains a conserved coiled-coil region (Figure 2B), also present in metazoans where it is responsible for interactions with other proteins, notably PKD1, ionic channels (e.g. TRPV4 and TRPC1), and cytoskeleton-related proteins (e.g. α-actinin and troponin) [28]–[32].

Two distinctive features of the human PKD2, the presence of an EF-hand domain and of a large region ensuring retention in the endoplasmic reticulum [33], are absent from the other PKD2 orthologs analyzed here (*C. elegans*, *D. melanogaster* and *Dictyostelium*) (Figure 2B). As the localization of the human ortholog is still a matter of debate – PKD2 has been localized to plasma membrane, primary cilia, ER, and Golgi [34], [35] – we decided to check where the *Dictyostelium* PKD2 ortholog was localized.

**Figure 3:**
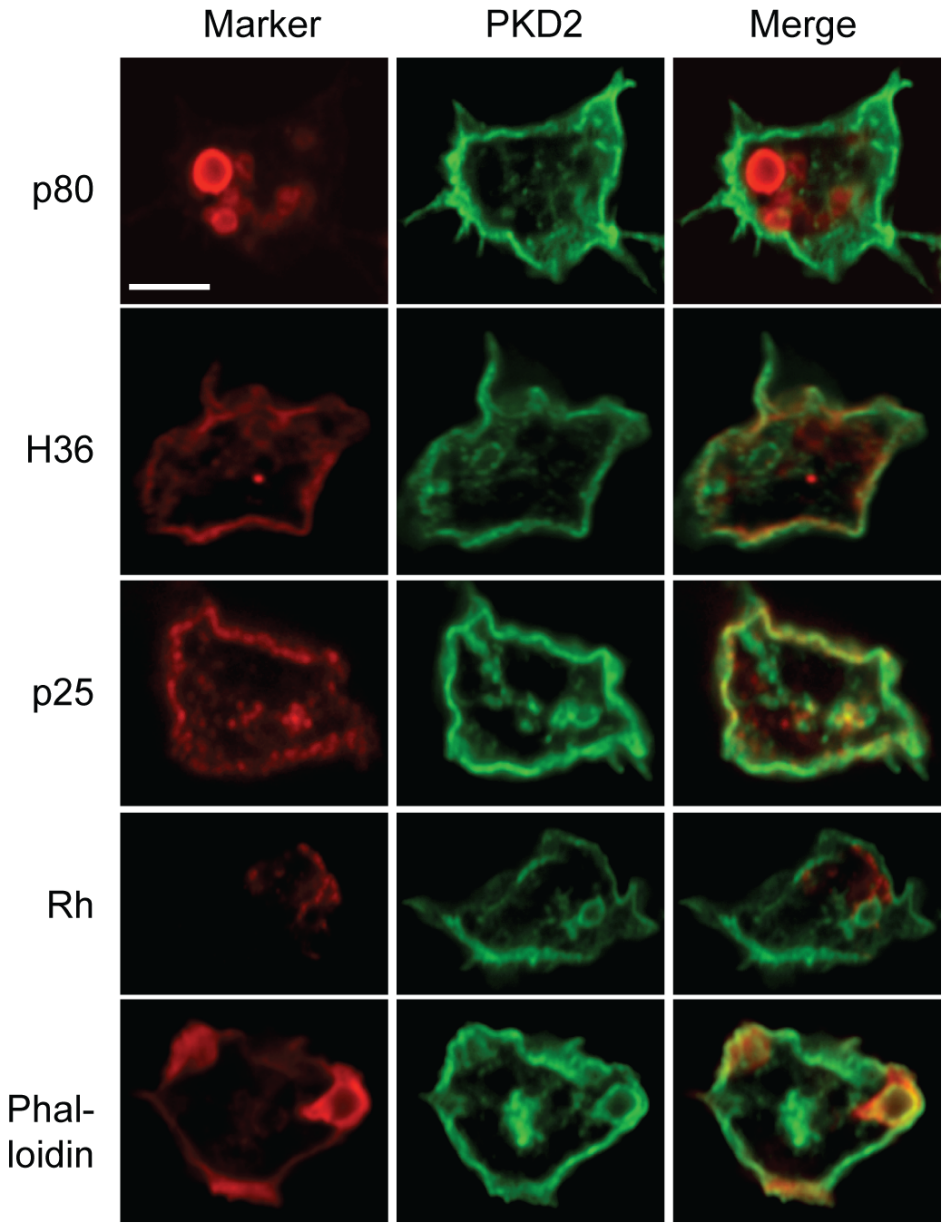
PKD2 localizes at the plasma membrane. Confocal images of PKD2-Flag transfected cells, labeled for the FLAG epitope (middle column, green) and cellular markers (left column, red) for late endosomes (p80), plasma membrane (H36), recycling endosomes (p25), contractile vacuole (Rh) and actin (phalloidin). Scale bar: 5 µm.

Protein localization was assessed by immunofluorescence using a Flag-tagged PKD2 construct (Figure 3). The majority of the protein was present at the plasma membrane, as shown by the extensive co-localization with a plasma membrane marker (H36). No significant co-localization was seen with a marker of late endosomal compartments (p80) or contractile vacuole (Rhesus). The internal structures in which PKD2 can also be detected co-localized partially with recycling endosomes (p25 marker) and with newly formed endosomes (actin-positive).

These observations suggest that in *Dictyostelium*, PKD2 is mostly localized at the cell surface and in early endocytic compartments. Given the surface localization of *Dictyostelium* PKD2, it seems reasonable to hypothesize that its major role in the response to mechanical stress is to mediate transient entry of extracellular calcium in response to mechanical signals.

### Role of PKD2 in calcium-stimulated lysosome exocytosis

**Figure 4:**
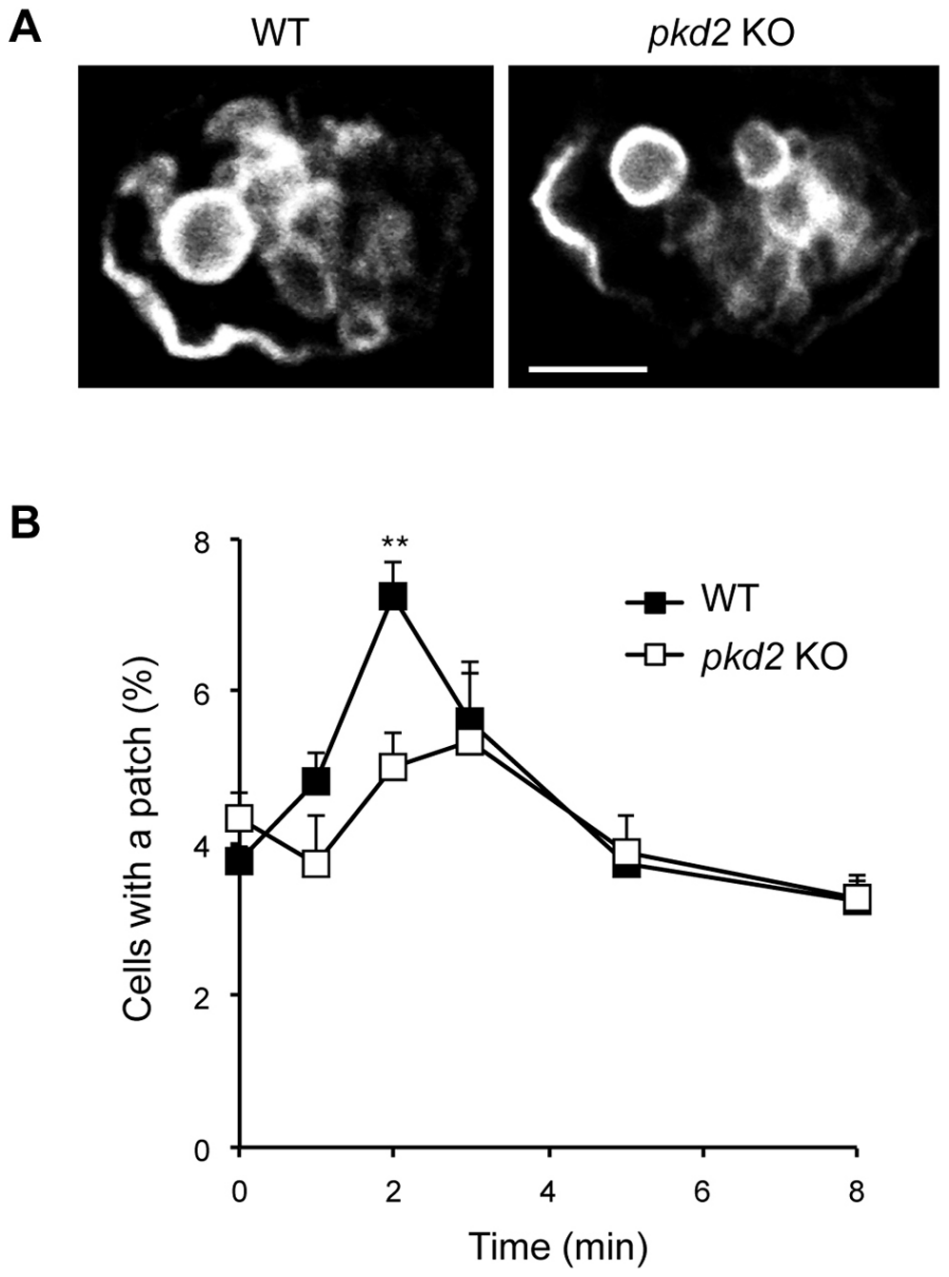
PKD2 is essential for calcium-induced lysosome exocytosis. A) Confocal image of typical exocytic p80 patches in WT and *pkd2* KO cells. Scale bar: 5 µm. B) Percentage of cells exhibiting an exocytic patch after transfer to a medium containing 1 mM CaCl_2_. WT cells showed a rapid and transient increase in fusion events, peaking after 2 minutes, while no induction of lysosome exocytosis was seen for *pkd2* KO cells. ** p<0.01, compared to WT values at each time point; n = 5.

Another cellular function directly linked to transient increases in cytosolic calcium is the secretion of lysosomes. In mammalian cells, lysosome exocytosis may be triggered by several different stimuli that promote rises in cytoplasmic calcium, including a sudden increase in extracellular calcium levels [36]–[38]. In *Dictyostelium*, secretory lysosomes are highly enriched in the endosomal p80 protein, and their fusion with the plasma membrane can be easily assessed by the formation of transient p80-rich microdomains, denominated exocytic patches (Figure 4A) [39]. In nutrient medium (containing approximately 30 µM calcium [24]), secretory lysosomes fuse constitutively with the cell surface. Consequently, 4.1±0.2% of WT cells exhibit an exocytic patch, and *pkd2* KO cells present a similar phenotype (5.1±0.5%).

When cells were exposed suddenly to a higher extracellular calcium concentration (1 mM), a burst of lysosome fusion was observed in WT cells, as shown by a rapid and transient 2-fold increase in the number of exocytic patches (Figure 4B). On the contrary, in the same conditions no increase in fusion of lysosomes with the cell surface was observed in *pkd2* KO cells (Figure 4B). Indeed for *pkd2* KO cells, the variations over time were not significantly different from the control values at time 0. This result suggests that PKD2 plays a role in calcium-induced lysosome secretion, probably by mediating a rapid influx of extracellular calcium.

### PKD2 is not involved in folate chemotaxis

**Figure 5:**
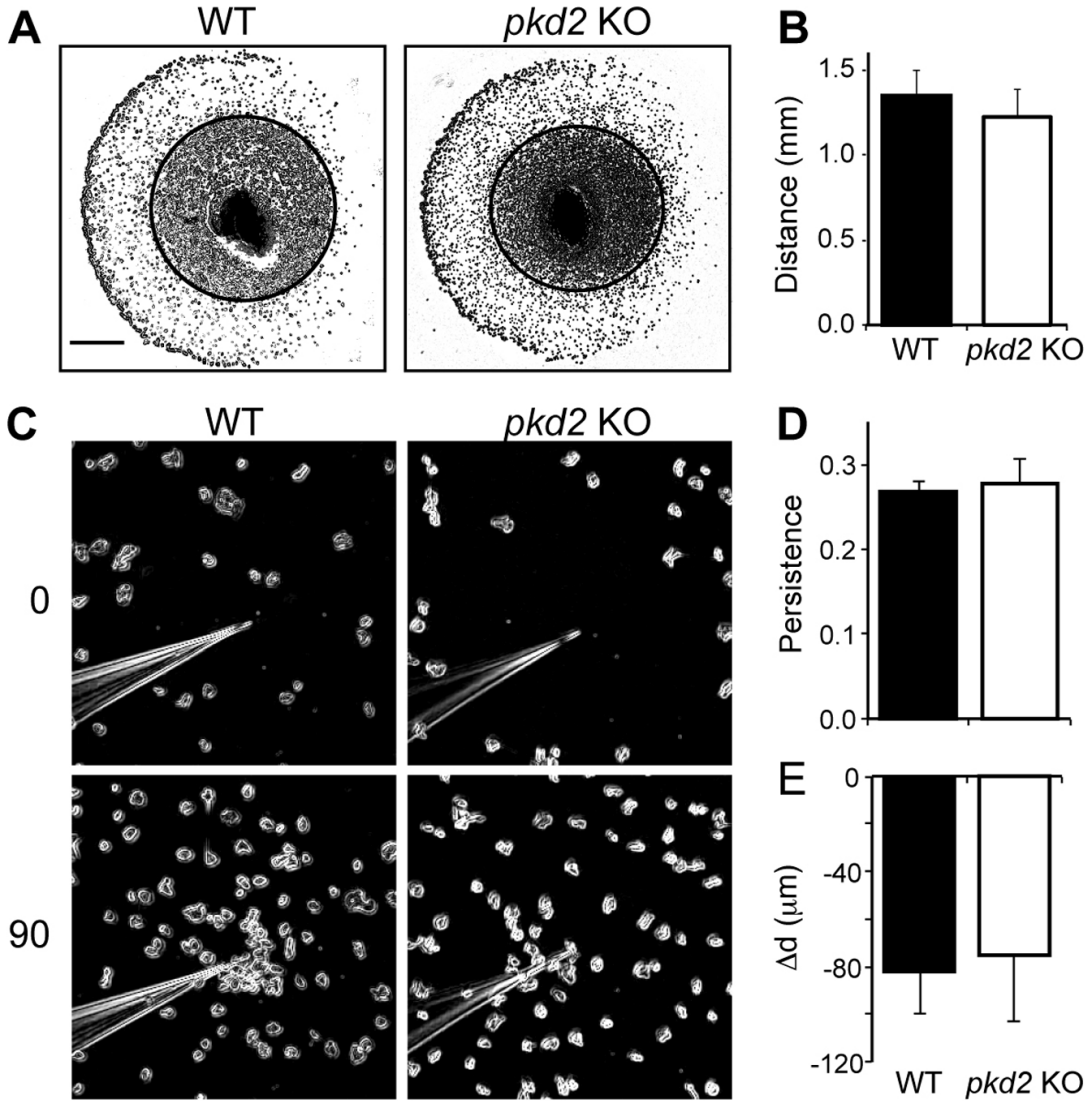
PKD2 is not essential for folate chemotaxis. A) Cells were deposited at the surface of buffered agar plates 4 mm away to a source of folate (to the left), and allowed to migrate for 5 h. Phase-contrast pictures of one representative experiment after migration are shown. The drawn inner circle represents the position of the cells at time 0. Scale bar: 1 mm. B) Distance travelled by the front of cells between times 0 and 5 h. WT and *pkd2* KO cells moved towards folate with similar efficiency; n = 4. C) WT and *pkd2* KO cells submerged in phosphate buffer were allowed to move for 90 min towards a micropipette emitting folate. Phase-contrast pictures of one representative experiment are shown at times 0 and 90 min. D) In the experimental setup described in (C), persistence of cell movement was measured as the distance from the initial to the final cell position divided by the total travelled distance. No significant difference was observed between WT and *pkd2* KO cells; n = 4. E) The distance of each cell to the micropipette tip was measured at time 0 and 90 min to calculate the overall migration towards the source of folate (Δd). Negative values indicate that cells are moving towards the folate source. No significant difference was observed between WT and *pkd2* KO cells; n = 4.

To evaluate if PKD2 was implicated in cell orientation and taxis in a more general manner, we analyzed the ability of vegetative cells to migrate towards folate. Chemotaxis assays were conducted either on an agar surface or in submerged conditions. Chemotaxis on buffered agar was assessed by spotting cells in close proximity to a folate source, and observing the ability of cells to move towards the chemoattractant after 5 hours. As can be seen in Figure 5A, both WT and *pkd2* KO cells were able to orientate towards folate: the front of cells moved to the left, where the folate source was located. The distance travelled by both cell types was also the same (Figure 5B).

Similarly, cells on phosphate buffer subjected to a folate gradient generated by a micropipette were able to move towards the source of folate, as denoted by the increased number of cells near the tip of the micropipette (Figure 5C). The directional persistence (measured as the sustained cell movement in the direction of the tip) was identical for WT and *pkd2* KO (Figure 5D). Similarly, the oriented displacement towards the pipette tip was the same in WT and *pkd2* KO cells (Figure 5E). Altogether, these results indicate that the PKD2 channel is not necessary for chemotaxis towards folate in *Dictyostelium*.

## Discussion

In this work, we showed by systematic comparative analysis of KO strains that in *Dictyostelium*, PKD2 is the most important protein for rheotaxis. Of all mutants analyzed, only *pkd2* KO cells were unable to respond to a flow-induced shear stress, and a WT phenotype was restored by complementation with a full-length PKD2. This is the first time that PKD2 has been implicated as a molecular player in mechanotaxis in *Dictyostelium*. Other potential candidates were also assayed for their role in shear-flow-induced cell motility, notably other calcium channels and orthologs of a bacterial mechanosensing channel (MscS) and of a metazoan integrin-beta (SibA). Of all these, only TRP-ML (or mucolipin) deficiency led to a significant, though limited, reduction in mechanosensing.

Previous studies have assessed the response of *Dictyostelium* cells after mechanical stresses caused by electric fields [23], compression [22], stretching [40] or a fluid flow [20], [21]. In all these studies, depletion of extracellular calcium completely abolished the response to stimuli, suggesting a role for calcium transporters in the process. In addition, gadolinium (Gd^3+^), a known blocker of plasma membrane calcium channels and stretch-activated channels, also impaired the response to mechanical stress [21], [23], [40]. Moreover, one of the hallmarks of the response to mechanical stress is an increase in cytosolic calcium, both in mammalian and *Dictyostelium* cells [6], [13], [23], [40]. However, it is a matter of debate if the calcium originates from the extracellular medium or from the intracellular stores (and, in consequence, if the major contribution for such response comes from intracellular or plasma calcium channels).

In the aforementioned studies, the potential role of the *Dictyostelium* IP_3_ receptor ortholog (*iplA*) in mechanosensing was assessed. Mammalian IP_3_ receptors are implicated in cellular calcium homeostasis by controlling calcium release from ER stores. In *Dictyostelium*, depletion of the *iplA* gene did not impair chemotaxis (either towards cAMP or folic acid) [41], [42] or the mechanotactic response to electric fields [23] or to flow-induced shear stress [21]. Most of these experiments were performed in the presence of an excess of extracellular calcium, a condition similar to that used in our study. It remains possible that in different conditions, notably when the extracellular calcium concentration is lower, release by IplA of intracellular stores of calcium may play a more critical role in mechanosensing, as suggested previously [21].

In summary, our observations are in agreement with previous results suggesting that mechanotaxis involves primarily a direct transfer of calcium from the extracellular medium to the cytosol. They further suggest that PKD2 may be the main effector of this calcium transport across the plasma membrane by showing that PKD2 is localized primarily at the cell surface in *Dictyostelium* and is a key element in mechanosensing. This hypothesis is reinforced by our observation that PKD2 is essential for calcium-induced exocytosis of secretory lysosomes (or post-lysosomes). Indeed, since we observe that calcium-induced lysosome secretion is PKD2-dependent and is maximal two minutes after raising the extracellular calcium concentration, it seems probable that lysosome secretion is caused by a direct transfer of calcium from the extracellular medium to the cytosol through PKD2. Unfortunately, we have been unable to measure cytosolic calcium levels in *pkd2* KO cells, either by using fluorimetric and ratiometric probes or with an aequorin genetic system (we were never capable of measuring a signal above background values). So, it remains to be seen if depletion of PKD2 channel really impairs entry of extracellular calcium, after a mechanical stimulus or after addition of extra calcium on the medium.

How does PKD2 open in response to mechanical stress? In mammalian cells, a number of proteins associated to PKD2 have been proposed to play a key role in its activation. In ciliated cells from the kidney and vascular endothelium, the PKD1/PKD2 complex has been implicated in mechanosensing [43], [44]. Other results have suggested that this complex does not act as a calcium channel, but rather regulates the function of other potential channels (as TRPV4 and TRPC1), potentially via interactions with cytoskeleton components such as filamin [17], [28], [32]. Remarkably, in *Dictyostelium*, PKD1 as well as TRP channels from the C and V families are absent, suggesting that PKD2 can act as a mechanosensor in the absence of other associated membrane proteins, or making use of an entirely different set of interacting partners. PKD2 may even act as a *bona fide* stretch-activated channel of *Dictyostelium*, ensuring both detection of the mechanical stress and calcium entry following activation. If new candidates implicated in mechanosensing are identified in various systems, the validity and the generality of these observations may be checked in *Dictyostelium* by generating the corresponding knockout strains and analyzing their phenotype.

## Materials and Methods

### Cells and reagents

The *Dictyostelium* strains employed here were all derived from the subclone DH1-10 [45] of the DH1 strain, referred to as wild-type (WT) for simplicity. Cells were grown in HL5 medium at 21°C and subcultured twice a week to maintain the cell density below 10^6^ cells/ml. Migration experiments were conducted using either phosphate buffer (2 mM Na_2_HPO_4_, 14.7 mM KH_2_PO_4_, pH 6.0), or MES buffer (50 mM MES, pH 6.0) when calcium was added to the medium.

KO vectors for *pkd2*, *mscS*, *iplA* and *tpc* disruption were constructed using a blasticidin-resistance cassette flanked by two gene segments (Table 1 and Figure S2). The PvuI-digested plasmid was introduced into WT cells by electroporation, transfected cells were selected in the presence of 10 µg/ml blasticidin and individual clones were screened by PCR (Figures S2B and S2C). Three independent KO clones for each gene were used in parallel in this study, with identical phenotypes. The *sibA* and *mcln* KO cell lines were described previously [24], [46]. *iplA* KO cell lines using Ax2 [42] and JH10 [47] as parental backgrounds have also been described previously, but were not employed during this study.

A PKD2-Flag expression vector was constructed by introducing a C-terminal Flag epitope (DYKDDDDK) in frame with the PKD2 coding sequence into pDXA-3C [48]. This plasmid was transfected into *pkd2* KO cells by electroporation, and transfected cells were selected in the presence of 10 µg/ml G418.

### Fluorescence microscopy

Immunofluorescence for PKD2 localization and for quantification of the number of exocytic p80 patches was performed as described previously [24], [49]. For measurement of calcium-induced lysosome exocytosis, 10^6^ cells were allowed to attach to glass coverslips in HL5-MES medium for 3 hs, then transferred to HL5-MES containing 1 mM CaCl_2_, incubated between 0 and 8 minutes as indicated, fixed with paraformaldehyde 4%, permeabilized with Triton X-100 (0.08%) and labeled with mouse monoclonal antibody anti-p80.

Mouse monoclonal antibodies against the late endosomal marker p80 (H161), the p25 marker of recycling endosomes, and the plasma membrane H36 protein, as well as a rabbit antiserum against the contractile vacuole marker Rh50 were described previously [50]–[53]. F-actin was labeled with TRITC-phalloidin (Sigma-Aldrich). Mouse monoclonal anti-Flag antibody (clone M2) was from Sigma-Aldrich, and fluorescent secondary goat anti-mouse or anti-rabbit IgG from Molecular Probes.

### Cell migration under shear-flow stress

For measuring cell motility under flow conditions, the experimental setup was adapted from Decave et al. and Mennesson et al [20], [54]. 10^6^ *Dictyostelium* cells were allowed to attach on glass coverslips (24×50 mm) for 30 min in MES buffer containing 1 mM CaCl_2_. Coverslips were assembled in a parallel plate laminar flow chamber (Immunetics, Cambridge, MA), and the chamber connected to input and output tanks (Figure S1). Flow rates were controlled by the differential height between both tanks, and shear stress values were deduced by using the formula σ = 6Dη/wh^2^, where D is the flow rate (14 ml/min), η the fluid viscosity (0.001 Pa·s), h the chamber height (250 µm), and w the chamber width (5.5 mm). Cells were subjected to a 4 Pa shear stress and imaged every 15 seconds during 10 min in a phase-contrast, wide-field inverted Zeiss Axiovert 100M, with a Plan-Neofluar 10× objective. The images were acquired with a Hamamatsu CCD cooled camera and assembled into a movie using Metamorph (Molecular Devices, Sunnyvale, CA). Particle tracking application for Metamorph was used to track the individual trajectories and the total distance travelled by at least 15 cells per experiment. Speed was calculated as total distance divided by total time. Persistence was estimated by the ratio of the net distance (from initial to final point) to the total distance. Net displacement on the X axis (Δx) is given by the sum of all displacements on the X axis.

### Folate chemotaxis

To evaluate chemotaxis towards folate, two different assays were employed. The first assay was done by depositing 1 µl of 5×10^7^ cells/ml on a phosphate agar plate, 4 mm away from a folate source (5 mM) and analyzing cell orientation after 5 h [55]. A black mark on the bottom of the petri dish allowed us to align pictures taken at different time points. The travelled distance was calculated by measuring the displacement of the cell front.

For the second assay, cells were incubated overnight in HL5 in the presence of 1 mM folate, washed in phosphate buffer, and allowed to adhere for 15 min in 43 mm petri dishes. A folate gradient was created with a micropipette (pressure of 20 hPa) filled with 250 µM folate (Fiedler et al, submitted), and cells were imaged every 20 seconds for 90 minutes. Cell tracking was done as described above. The distance to the micropipette (d) was measured as the final distance of the cell to the micropipette minus the initial distance to the micropipette (cells moving towards the micropipette show negative Δd values, while cells moving randomly have Δd≅0).

### Sequence and phylogenetic analysis

Sequence similarity analyses were performed using BlastP program against the protein databases deposited at NCBI server. For phylogenetic analysis, protein sequences were aligned with CLUSTALX 2.0 [56] and maximum likelihood trees were done with MEGA 5.0 (WAG+F model, and parameters for invariable sites and gamma-distributed rate heterogeneity) [57]. One hundred bootstrap replicates were executed and bootstrap values drawn up on the consensus tree.

### Statistical analysis

Unless otherwise specified, for quantified data, the values represent the arithmetical mean and s.e.m. (the number of independent experiments is indicated by n). Statistical comparisons were done with student t-tests (two-tailed, unpaired).

## Supporting Information

Figure S1 **Shear-flow stress assay diagram.** In (A), schematic diagram of the chamber used for shear-flow stress experiments. A coverslip (in which cells were previously adhered for 30 min) is placed over two O-ring gaskets, and held in place by vacuum pressure. Buffer passes through the system via the in- and outlet openings; the speed of fluid flow is controlled by the height difference between the input and output tanks. In (B), the values for flow rate (black squares, in ml/min) and shear force (open circles, in Pa) are given in function of the height difference (in cm). A height difference of 30 cm was chosen for the experiments (corresponding to an applied force of 4 Pa).
(TIF)

Figure S2 **Generation of KO cells.** In (A), schematic representation of polycystin-2 (PKD2) gene in WT and *pkd2* KO cells (in the later, a blasticidin-resistance cassette was inserted via homologous recombination). Arrows indicate the position of the oligonucleotides (B) used to construct the KO vector (A) and to screen *pkd2* KO cells (C). In (B), gene position refers to position on the genomic sequence of the gene. Screen for *pkd2* KO cells was done by PCR, and different pairs of oligonucleotides were used to screen for gain or loss of signal in KO cells (C). In (D), 5′ and 3′ gene fragments used for generation of *iplA*, *mscS*, *pkd2* and *tpc* KO cells by homologous recombination. Screening was done exactly in the same way for the four KO cell lines.
(TIF)

Movie S1 **WT cells moving randomly, without any flow passing through the system.** Phase-contrast images were taken every 15 sec, during 10 min. Size: 160×95 µm.
(AVI)

Movie S2 **WT cells under shear-flow stress (4 Pa, from right to left).** Phase-contrast images were taken every 15 sec, during 10 min. Size: 160×95 µm.
(AVI)

Movie S3 ***pkd2*** **KO cells moving randomly, without any flow passing through the system.** Phase-contrast images were taken every 15 sec, during 10 min. Size: 160×95 µm.
(AVI)

Movie S4 ***pkd2*** **KO cells under shear-flow stress (4 Pa, from right to left).** Phase-contrast images were taken every 15 sec, during 10 min. Size: 160×95 µm.
(AVI)
