# Correction: Role of PKD2 in Rheotaxis in *Dictyostelium*

**DOI:** 10.1371/journal.pone.0091457

**Published:** 2014-02-28

**Authors:** The PLOS ONE Staff

Grants to the second and third authors (AV and JP) are incorrectly omitted from the Funding statement. The Funding statement should read: “The PC lab was supported by the Swiss National Foundation for Scientific Research (grant 31003A-135789), the Doerenkamp-Zbinden Foundation and the Fondation Egon Naef pour la Recherche in Vitro. The JP lab was supported by grants from the Swiss National Science Foundation. WCL was partially funded during this project with a Telethon Action Suisse grant and AV was funded by an EMBO Long Term Fellowship. The funders had no role in study design, data collection and analysis, decision to publish, or preparation of the manuscript.”
