# Supplementary figures and images for: Role of PKD2 in Rheotaxis in Dictyostelium
Source: PLoS One. 2014 Feb 10;9(2):e88682. doi: 10.1371/journal.pone.0088682 (PMC3919814; doi:10.1371/journal.pone.0088682)

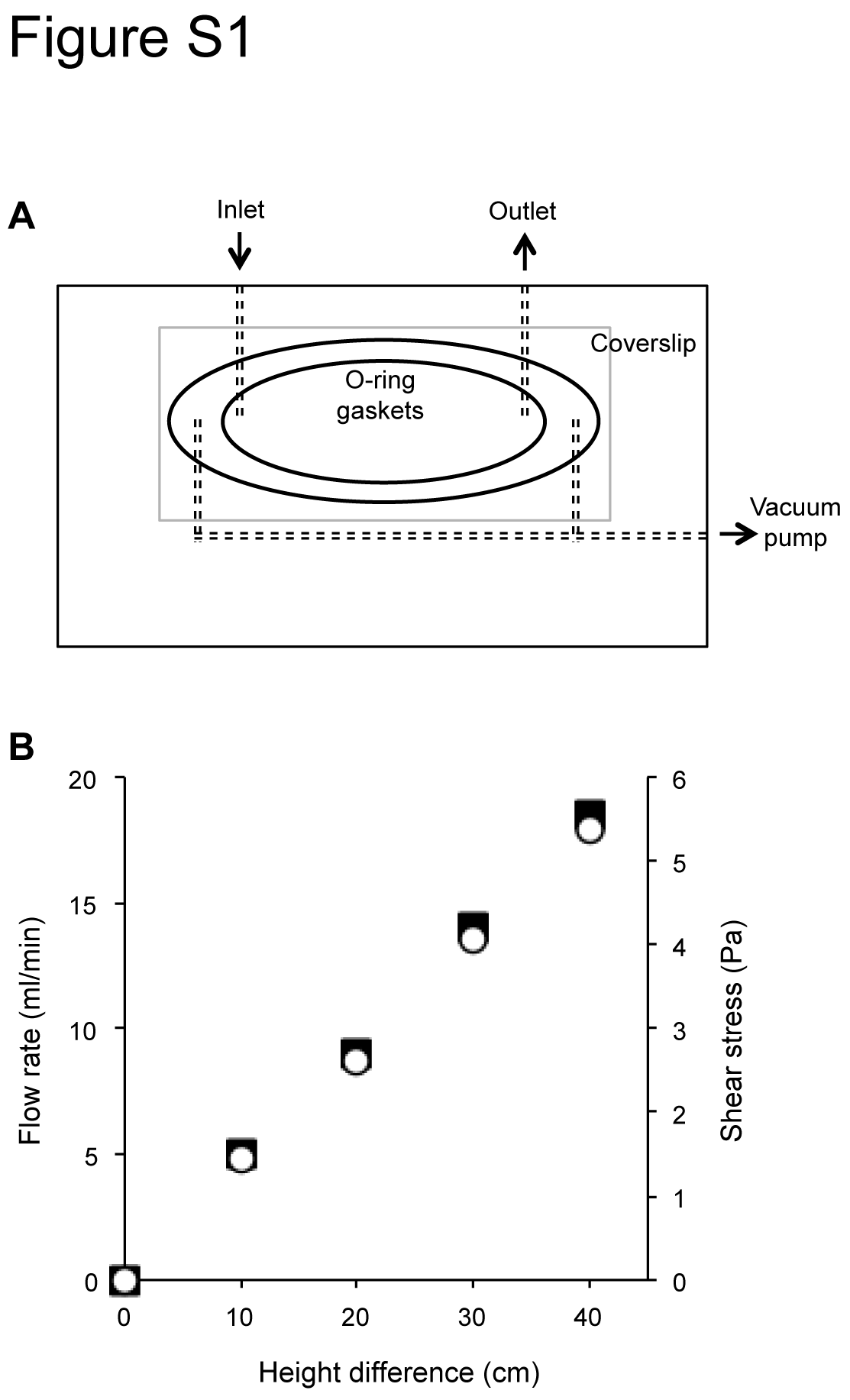

Supplement: Figure S1 — Shear-flow stress assay diagram. In (A), schematic diagram of the chamber used for shear-flow stress experiments. A coverslip (in which cells were previously adhered for 30 min) is placed over two O-ring gaskets, and held in place by vacuum pressure. Buffer passes through the system via the in- and outlet openings; the speed of fluid flow is controlled by the height difference between the input and output tanks. In (B), the values for flow rate (black squares, in ml/min) and shear force (open circles, in Pa) are given in function of the height difference (in cm). A height difference of 30 cm was chosen for the experiments (corresponding to an applied force of 4 Pa). (TIF) [file pone.0088682.s001.tif]

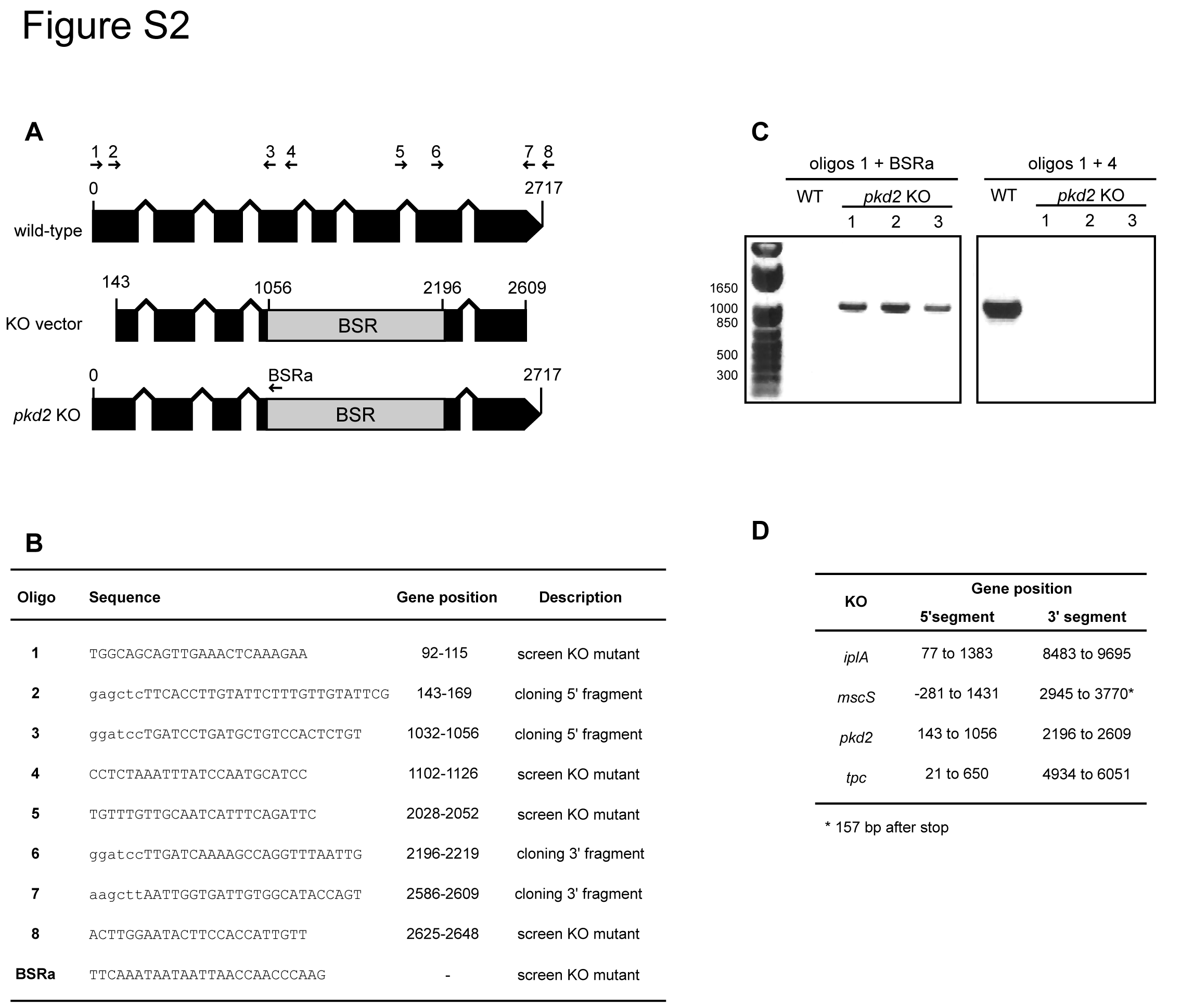

Supplement: Figure S2 — Generation of KO cells. In (A), schematic representation of polycystin-2 (PKD2) gene in WT and pkd2 KO cells (in the later, a blasticidin-resistance cassette was inserted via homologous recombination). Arrows indicate the position of the oligonucleotides (B) used to construct the KO vector (A) and to screen pkd2 KO cells (C). In (B), gene position refers to position on the genomic sequence of the gene. Screen for pkd2 KO cells was done by PCR, and different pairs of oligonucleotides were used to screen for gain or loss of signal in KO cells (C). In (D), 5′ and 3′ gene fragments used for generation of iplA, mscS, pkd2 and tpc KO cells by homologous recombination. Screening was done exactly in the same way for the four KO cell lines. (TIF) [file pone.0088682.s002.tif]
